# Rituximab Monotherapy in the Management of a Rare Case of an HIV Associated Lymphoproliferative Disorder

**DOI:** 10.1155/2017/5235163

**Published:** 2017-04-30

**Authors:** Jason Hew, Fauzia Rana, Louise Zhou

**Affiliations:** Department of Internal Medicine, Division of Medical Oncology, University of Florida COM, Jacksonville, FL, USA

## Abstract

*Background.* Castleman's disease (CD), also known as angiofollicular node hyperplasia, is a rare heterogenous lymphoproliferative disorder. This disease exists as two distinct entities: a localized or unicentric CD (UCD) which has a more benign clinical course and multicentric CD (MCD) which is a systemic disease and carries a worse prognosis. MCD is often associated with human immunodeficiency virus (HIV) infection and these patients are usually coinfected with human herpes virus-8 (HHV-8). Rituximab is an anti-CD20 monoclonal antibody that has become integral to the management of this disease. It is used alone or in combination with chemotherapy to treat MCD.* Case Report.* We describe a case of a 58-year-old man with HIV and HHV-8 MCD and evidence of organ failure with a poor performance status that went into complete remission after four cycles of therapy with weekly rituximab.* Conclusion.* HIV-MCD can be challenging to diagnose and to manage. Early recognition can reduce morbidity and mortality associated with the disease. Rituximab monotherapy can be used as a safe and effective treatment option in patients with a poor performance status.

## 1. Background

CD was first described in 1956 at the Massachusetts General Hospital by the pathologist Benjamin Castleman [1, 2]. The initial cases described were localized or UCD, and it was thought to be a nonneoplastic disorder which presented as a mediastinal mass in young adults [1]. MCD is an aggressive disease which usually presents in the sixth decade of life and if left untreated is associated with a high risk of mortality [3]. Clinically, patients can present with generalized lymphadenopathy, hepatosplenomegaly, fevers, and night sweats. The diagnosis of MCD requires pathological confirmation typically through an excisional lymph node biopsy [1, 4]. In addition, patients should have fever, elevated C-reactive protein, and other signs of active disease. MCD has three major histological subtypes; these are hyaline vascular, plasma cell, and mixed variants. The plasma cell variant is the most common subtype of MCD seen in up to 80–90% of cases while the hyaline vascular subtype occurs more frequently in UCD [1].

Most of the cases of MCD are associated with HIV infection and these patients are universally coinfected with HHV-8 [1]. Although the pathogenesis of MCD is not well understood, it appears that HHV-8 has a key role in the development of this disease. HHV-8 is a gamma herpes virus which has been linked to acquired immunodeficiency syndrome (AIDS) defining illnesses such as Kaposi sarcoma (KS) as well as primary effusion lymphoma (PEL) [4]. The presence of HHV-8 is known to cause cytokine dysregulation leading to excessive release of interleukin-6 (IL-6). This occurs particularly during viral replication where the HHV-8 infected B-cells release the virally encoded form of IL-6 (vIL-6) that subsequently activates the human IL-6 receptor which causes the systemic inflammatory response seen in MCD. Although both MCD and KS are associated with the presence of HHV-8, elevations of vIL-6 are rarely seen in KS [1, 4, 5]. Since the advent of highly active retroviral therapy (HAART), the incidence of KS has declined while the MCD incidence has risen from 2.3 per 10,000 patient-years in the pre-HAART era prior to 1996 to 8.3 per 10,000 patient-years since the year 2000. There also seems to be no direct correlation between the degree of immunodeficiency or Cluster of Differentiation 4 (CD4) count and the incidence of MCD [6, 7].

Optimal management strategies for MCD are not well defined and clinical practice varies. To date, there has only been one randomized clinical controlled trial evaluating treatment in MCD. Due to the rarity of this disease, the majority of the data comes from systematic literature reviews, case series, and case reports. Based on several published reports, high rates of response are seen with the use of rituximab by itself or in combination with chemotherapy in HIV-MCD. This case highlights and confirms the efficacy of this drug [7, 8].

## 2. Case

**Figure 1:**
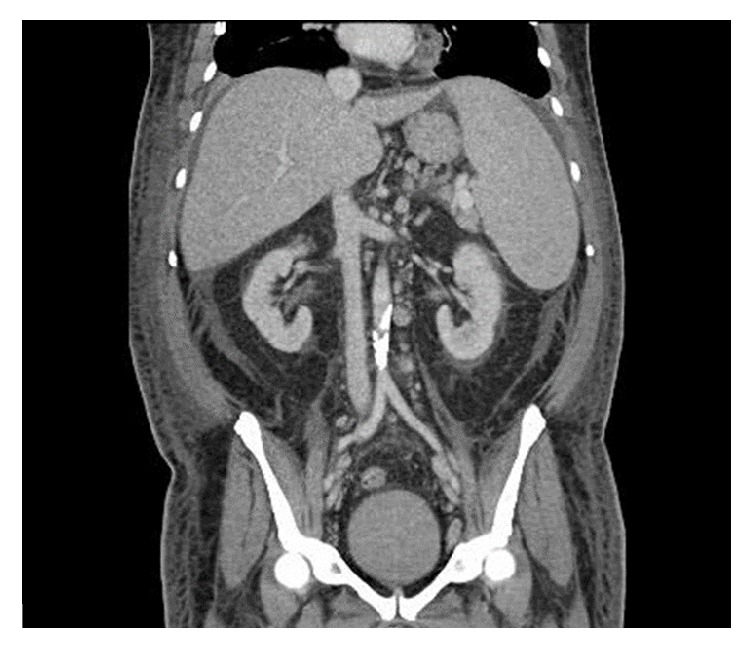
Pretreatment CT with contrast showing enlarged intra-abdominal nodes and splenomegaly.

A 58-year-old Caucasian man presented with a one-month history of fever, night sweats, progressive fatigue, dyspnea, abdominal pain, and swelling. He had a past medical history of chronic hepatitis C and HIV disease/AIDS which was found 8 months prior to his presentation. He was started on HAART prior to this hospital visit and had a CD4 count of 83 cells/*μ*L and a viral load of 214 copies/mL at the time of his presentation. His initial examination findings were significant for a middle-aged Caucasian male in moderate distress that was tachycardic at rest with a heart rate of 110 beats/min and febrile with a temperature of 101.6°F with generalized peripheral adenopathy, anasarca, and splenomegaly and decreased breath sounds in bilateral lung bases. Initial laboratory investigations revealed that he was pancytopenic with hemoglobin of 6.7 g/dL, white blood cell (WBC) count of 2700/*μ*L, and a platelet count of 64000/*μ*L. His complete metabolic panel showed that he was hypoalbuminemic with albumin of 2.3 g/dL; the remainder of his panel was within normal limits. Computerized Tomography (CT) imaging was performed on his chest, abdomen, and pelvis with contrast which showed diffuse bulky adenopathy, marked splenomegaly with evidence of portal hypertension, moderate ascites, and bilateral pleural effusions (Figure 1). The initial management was directed at treating him for possible sepsis and he was placed on broad-spectrum empiric antibiotics which did not improve his symptoms and blood and urine cultures returned negative.

A core needle biopsy was performed on an enlarged left axillary lymph node on the third day of hospitalization which demonstrated features consistent with MCD with focal features of early development of a plasmablastic microlymphoma. The pathology report highlighted the presence of numerous plasma cells seen mainly in the mantle zone and interfollicular area showing nuclear HHV-8 positivity. The plasma cells were evaluated using mRNA in situ hybridization (ISH) studies which were polyclonal for kappa/lambda. CD staining was performed which demonstrated a background of T-cells with occasional microscopic foci of lambda restricted plasmablast (HHV-8+), variably expressing CD20 seen invading follicles. A unilateral bone marrow aspirate and biopsy was subsequently done which showed a hypercellular marrow with evidence of hemophagocytosis in a background of plasma cell infiltration consistent with involvement by MCD. Additional laboratory studies revealed markedly elevated C-reactive protein (CRP) of 229 mg/L (reference range: <5 mg/L) and an elevated lactate dehydrogenase (LDH) and *β*2 microglobulin which were 282 U/L (reference range: 126–266 U/L) and 9.7 mg/L (reference range: 0.6–2.4 mg/L), respectively. His serum protein electrophoresis showed hypergammaglobulinemia and hepatitis B was found to be negative.

Prior to starting treatment, this patient was evaluated for skin lesions suggestive of Kaposi's sarcoma and no lesions were identified. He was also started on treatment with ganciclovir for HHV-8 but this was subsequently discontinued due to worsening cytopenias. His laboratory studies suggested worsening of his organ function since his admission with a decrease in his platelet count to 10000/*μ*L; his albumin was further decreased to 1.7 g/dL; both his creatinine and his bilirubin had risen from previous normal values to 1.39 mg/dL and 1.5 mg/dL, respectively. He required multiple transfusions while in hospital and had a total of 9 units of packed red blood cells and 4 units of platelets. Given his declining performance status (PS) which was considered to be Eastern cooperative oncology group PS grade 3 (ECOG PS grade 3), he was not considered to be a candidate for chemotherapy and was offered treatment with rituximab only.

**Figure 2:**
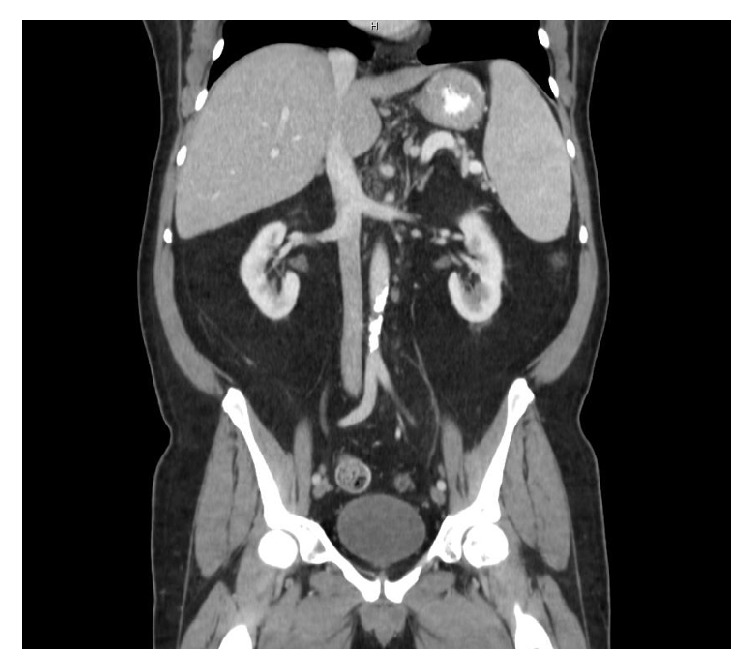
Posttreatment CT with contrast of the abdomen showing resolution of the enlarged intra-abdominal lymph nodes and reduction in splenic size.

On day 10 of his hospitalization, treatment with rituximab was initiated at 375 mg/m^2^ with plans for weekly administration for a total of 4 cycles. This patient tolerated treatment with rituximab without any adverse effects. Within days of completing therapy, there was an observed clinical response with the resolution of his fever, night sweats, and anorexia as well as rapid improvement of his cytopenias. He completed all 4 cycles of rituximab in hospital. His posttreatment CT with contrast of the abdomen showed a marked reduction in the size of his enlarged intra-abdominal lymph nodes and splenic size (Figure 2). A follow-up PET (positron emission tomography)/CT study was done 2 months after completing therapy with rituximab which showed complete resolution of his previously seen bulky adenopathy and pleural effusions. Clinically, his performance status improved to ECOG PS grade 0 with no limitations in his activity level. Laboratory studies demonstrated resolution of his pancytopenia and hypoalbuminemia. Approximately 8 months after completing his treatment with rituximab, he remains in clinical remission.

## 3. Discussion

MCD continues to present a challenge for clinicians despite being discovered over fifty years ago. Ideal management strategies are not known and there is still a limited understanding of its pathogenesis [3, 7]. In the era of HAART, the incidence of HIV associated MCD continues to rise while other HHV-8 associated diseases are now seen less frequently. Patients with HIV-MCD tend to present more often with fulminant disease as compared to their idiopathic counterparts and can be a diagnostic dilemma at the time of presentation. As seen in this case, delays in making the diagnosis and initiating management may prove to be costly to patients as this disease can be rapidly progressive and even fatal. Clinicians should still evaluate these patients for opportunistic infections and lymphomas which may present similarly and can coexist with MCD [1, 3, 7].

Clinical practice in the management of HIV-MCD varies, but there appears to be a growing body of evidence supporting the use of rituximab by itself or in combination with chemotherapy. Rituximab is a monoclonal antibody directed against CD20 positive cells. Although the HHV-8 infected plasmablasts do not highly express CD20, it has been suggested that this drug is effective in eliminating the affected B-cells in the mantle zone of the lymph node and this decreases the inflammatory response and reduces the HHV-8 viremia. Two pilot trials have been completed which show the benefit of rituximab in HIV-MCD. Gerard et al. conducted a phase II open label study evaluating the effect of rituximab given at 375 mg/m^2^ weekly for 4 weeks on 24 stable patients with HIV-MCD previously maintained on single agent chemotherapy. 17 of 24 patients (71%) were alive in sustained remission at day 365 without specific treatment, and the overall survival rate was 92% (95% CI: 71% to 98%). There were no severe adverse effects reported [7]. Three of five patients however had flares of their Kaposi's sarcoma but did not require additional treatment. Similarly, in another open label phase II study by Bower et al., rituximab was shown to be efficacious. This study enrolled 21 treatment naive patients with HIV-MCD to receive 4 weeks of weekly rituximab, The results showed overall and disease-free survival rates at 2 years of 95% (95% CI: 86% to 100%) and 79% (CI: 49% to 100%), respectively. The main adverse effect reported was reactivation of Kaposi's sarcoma [4, 7, 9]. Other reports of prolonged remission have been seen when rituximab has been combined with chemotherapy [7, 9, 10]. In a retrospective multicenter analysis evaluating therapy in HIV-MCD conducted by Hoffman et al., there was a statistically significant survival benefit observed with using rituximab based therapy versus chemotherapy and antiviral therapy only [10].

Alternative treatment options have been used in the management of HIV-MCD often with temporary and partial results. Glucocorticoids have been reported to be effective in providing symptom control and short-term responses [1, 7]. Historically, prior to the introduction of rituximab, single agent chemotherapy and combination chemotherapy regimens were used such as cyclophosphamide/vincristine/doxorubicin and prednisone (CHOP) and other lymphoma regimens with more durable responses being reported with the use of combined agents [1–3]. Antiherpes virus agents have been explored as a treatment option that would target HHV-8, the implicated pathogen in this disease during its lytic replication. Uldrick and colleagues conducted a pilot study on 14 patients with HIV-MCD who were treated with a combination of zidovudine and valganciclovir. The results were that 86% of these patients attained a clinical response and the median progression-free survival was 6 months. Another observation noted in the study was that reductions in the HHV-8 viral load, IL-6, and CRP levels correlated with an improved clinical response. Bortezomib, a drug frequently employed in the management of multiple myeloma, has also been reported anecdotally to be efficacious in the management of MCD and in some instances provided a durable response. Bortezomib is a proteasome inhibitor which acts on plasma cells and is believed to decrease the production of IL-6 through the nuclear factor kappa blockade [1, 7].

At present, there has only been one randomized double blinded placebo control trial evaluating drug therapy in MCD. Siltuximab which is a monoclonal antibody to the IL-6 receptor was tested in 79 patients with HIV negative, HHV-8 negative MCD compared to placebo. The primary endpoint of the study was a durable radiological and clinical response lasting at least 18 weeks. Treatment with siltuximab was associated with a higher rate of achieving the primary endpoint (34% versus 0%). Radiographic tumor response rates were seen in 38% and 4% of patients, favoring the siltuximab arm, and symptomatic response rates were 57% and 19% [11]. Siltuximab has been US Food and Drug Administration (FDA) approved for the treatment of HIV and HHV-8 negative MCD based on the results of this study. Tocilizumab which is a humanized antibody to the IL-6 receptor has also been shown to be active in the management of HIV negative MCD in a phase 2 trial; however, it has not been approved for use in the treatment of MCD. It is uncertain whether IL-6 receptor antibodies will provide similar results in patients with HIV-MCD [1, 7, 12]. Nagao et al. reported on 2 patients with HIV-MCD who were treated with tocilizumab and received rapid but short-term benefit from this treatment with relapses occurring at 15 and 22 weeks. These patients were subsequently treated with rituximab and achieved durable and complete remission [12].

## 4. Conclusion

MCD continues to be a rare lymphoproliferative disorder despite a rising incidence in HIV patients. There is no well-defined treatment strategy and clinical practice varies. The HIV-MCD patient is clinically challenging as these patients can present with fulminant disease. The management of immunodeficiency as well as concomitant opportunistic infections and other lymphomas needs to be incorporated in the treatment plan. Rituximab appears to be safe, effective, and less toxic than traditional cytotoxic chemotherapy and can result in durable remission. It is not known whether rituximab as a single agent is as effective as when it is combined with chemotherapy. There remain several unanswered questions with regard to the treatment of MCD and further studies are warranted.
